# Surgical excision of a malignant metastatic melanoma located in a skeletal muscle of the lateral thorax of a horse

**DOI:** 10.1002/vms3.366

**Published:** 2020-09-29

**Authors:** Theodora Billi, Vasiliki Karadima, Panagiota Tyrnenopoulou, Emmanouela P. Apostolopoulou, Georgia D. Brellou, Nikolaos Diakakis

**Affiliations:** ^1^ Faculty of Health Sciences Equine Unit School of Veterinary Medicine Aristotle University of Thessaloniki Thessaloniki Greece; ^2^ Faculty of Health Sciences Laboratory of Pathology School of Veterinary Medicine Aristotle University of Thessaloniki Thessaloniki Greece

**Keywords:** equine, melanoma, skeletal muscle, thoracic

## Abstract

**Background:**

A 20‐year‐old grey Warmblood gelding that had history of dermal melanomatosis.

**Objective:**

To report surgical treatment of a 6‐cm large malignant metastatic melanoma located in the cutaneous trunci muscle of the left lateral thorax of a horse.

**Methods:**

A 20‐year‐old grey Warmblood gelding was referred for evaluation of a large, rapidly growing, thoracic mass. Clinical examination revealed an ovoid, firm, non‐painful, movable mass, approximately 6 cm in diameter, located in the cutaneous trunci muscle of the left lateral thorax. Multiple melanocytic nodules were also found at the perianal region and ventral tail. Rectal examination, ultrasonography and endoscopy of the respiratory tract revealed no melanomas internally. Haematological and biochemical values were within normal limits. Surgical excision of both the thoracic mass and perianal nodules was the treatment of choice. Histopathology of the distant thoracic mass confirmed the diagnosis of malignant metastatic melanoma secondary to the perineal lesions that were confirmed as dermal melanomatosis.

**Results:**

The horse recovered uneventfully. Up until 3 years post surgery there was no tumour regrowth at the excision sites.

**Conclusion:**

Surgical excision of the metastatic melanoma was performed and considered successful, with no gross evidence of tumour reoccurrence. Despite the fact that surgical excision is considered a treatment option regarding equine dermal melanomatosis, there is no previously published study proposing surgical removal of distant malignant metastatic melanocytic masses. This is the first report of a successful surgical excision of a large (6 cm) metastatic melanocytic tumour from the skeletal muscle of the lateral thorax of a horse.

## Graphical abstract

A 20‐year‐old grey Warmblood gelding was referred for evaluation of a large, rapidly growing, thoracic mass. Histopathology of the distant thoracic mass confirmed the diagnosis of malignant metastatic melanoma. Surgical excision of the metastatic melanoma was performed and considered successful, with no gross evidence of tumour reoccurrence.This report proposes surgical excision as a treatment option for distant malignant metastatic melanocytic masses in skeletal muscle.

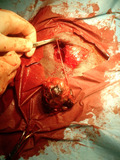

## 1 INTRODUCTION

Equine melanoma is one of the most common neoplasms reported in equine practice. It affects over 80% of aged grey horses (MacFadyean, 1933), occasionally non‐pigmented horses (white/albino or cremello) and rarely horses of other colour (Knottenbelt, 2015). Melanomas appear as slow growing, usually heavily pigmented, firm, solitary or multiple, discrete to coalescing, subcutaneous, locally invasive masses. Although equine melanocytic tumours can arise anywhere in the body, most commonly they originate at the skin of the ventral tail, the perineal region and external genitalia (Johnson, 1998). According to their clinical and histological features, they have been classified into four identifiable forms (melanocytic nevus, dermal melanoma, dermal melanomatosis and anaplastic malignant melanoma) (Valentine, 1995), whereas regarding their increasing expression of malignant behaviour, into five stages (Moore et al., 2012). Cutaneous melanomas may be benign or eventually progress and become malignant, spreading via lymphatics, blood, local translocation or a combination of these routes (Knottenbelt, 2015).

Various treatment options have been described regarding melanocytic tumours, but yet none is uniformly accepted. Although surgical excision is considered to be curative for melanocytic nevi and dermal melanoma, it has been reported as a controversial option for treatment of dermal melanomatosis due to its increased metastatic rate (Valentine, 1995). However, Groom and Sullins (2017) reported a study of 38 cases, where surgical excision of large (≥4 cm) single and coalescing melanomas was locally curative, referring only to the removed lesions.

The aim of this report is to describe a case of successful surgical removal of a large (6 cm) metastatic malignant melanocytic tumour of stage four, located in the cutaneous trunci muscle of the left lateral thorax.

## 2 CASE HISTORY

A 29‐year‐old grey Warmblood gelding (550 kg) was presented for evaluation and treatment of suspected melanocytic nodules at the perianal region and the ventral surface of the tail as well as for examination of a rapidly growing, 6 cm large mass at the lateral thorax.

Three years prior to referral, the owner had noticed multiple nodular lesions around the horse's anus and the base of the tail, as well as signs of dyschezia. A definitive diagnosis of dermal melanomatosis had been histologically confirmed and tumour masses had been surgically removed with the horse in standing position. Post‐operatively, the gelding had been unsuccessfully treated with oral cimetidine (2.5 mg/kg bwt TID) for 4 months. New tumours had developed near the excision sites and two additional surgeries, with one‐year intervals, were necessary for the alleviation of clinical sings. Three weeks prior to presentation the owner had noticed a growing mass at the thoracic musculature.

## 3 CLINICAL SIGNS

**FIGURE 1:**
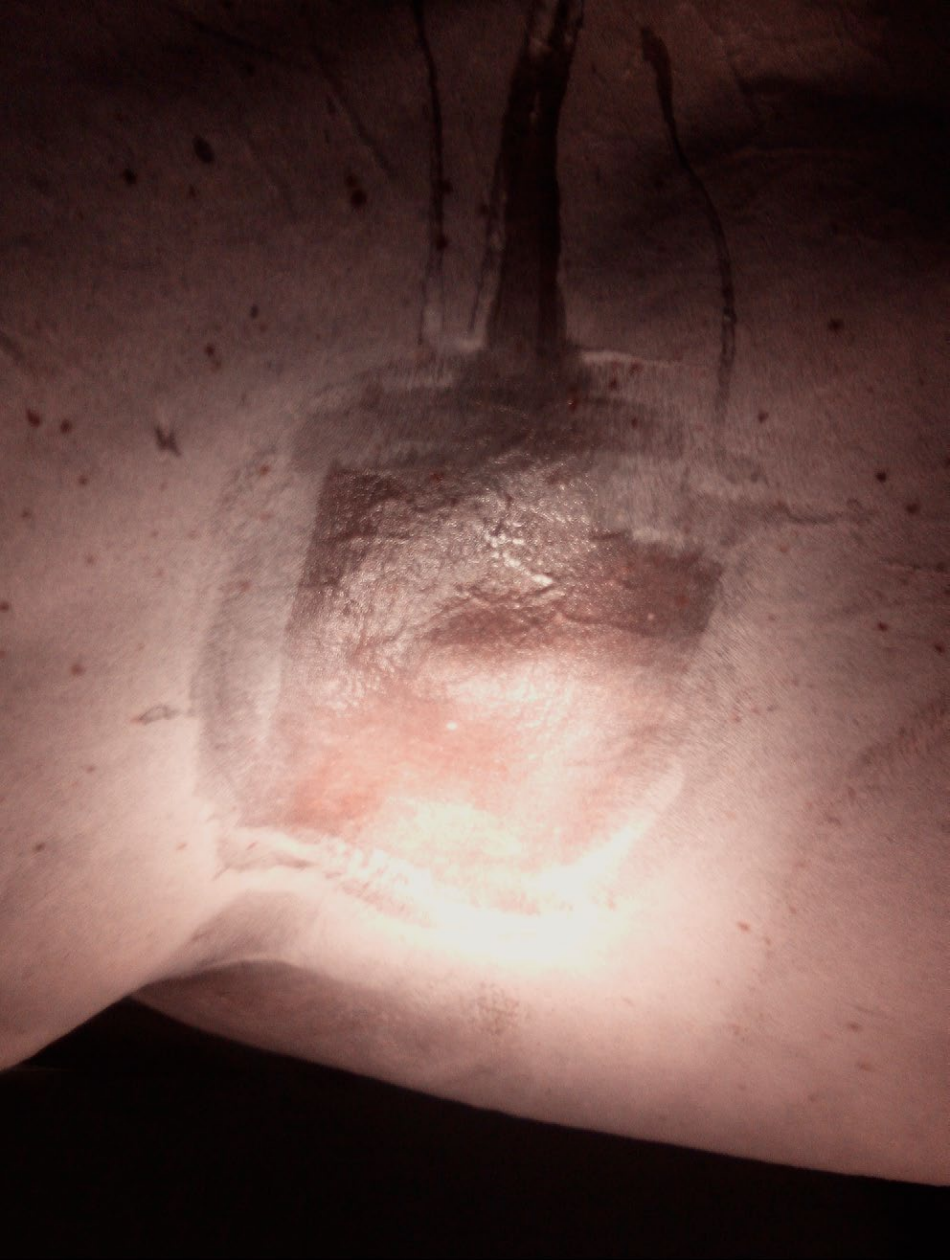
Tumour mass located in the musculature of the left lateral thorax, 10 cm dorsal to the olecranon and 5 cm caudal to the caudal border of the scapula. This picture was taken just before surgery

Upon clinical presentation, the horse was bright, alert, responsive and in good condition (body condition score 7/9). Clinical and haematological parameters were within normal limits. An ovoid, firm, well‐circumscribed, non‐painful, movable mass, measuring 6 cm × 5 cm × 4 cm, was palpable in the musculature of the left lateral thorax, 10 cm dorsal to the olecranon and 5 cm caudal to the caudal border of the scapula (Figure 1). Additional multifocal, spherical or ovoid cutaneous neoplastic lesions, of different diameter (0.5–3 cm), were observed around the anus and ventral tail. Rectal examination, ultrasonography of the abdominal and thoracic cavity and endoscopic examination of the upper respiratory tract revealed no abnormalities.

## 4 TREATMENT

Considering the specific location of the distant thoracic mass and the extensive growth of the perianal lesions, simultaneous surgical excision at both sides under general anaesthesia was elected.

The horse was anaesthetised and placed in right lateral recumbency with the thoracic mass uppermost. The left side of the thorax was clipped and aseptically prepared in a routine fashion.

Masses on the ventral tail were evaporated with the use of carbon dioxide (CO_2_) laser and were allowed to heal by second intention.

At the perianal region, a total of two incisions were made around the melanoma nodules, dorsal and lateral to the anal orifice using a No10 surgical blade. The nodules where sharply resected. Each incision was closed in a cruciate suture pattern using a No0 absorbable polyglactin suture. Surgical excision was performed only to those masses that caused physical obstruction of the anal orifice.

**FIGURE 2:**
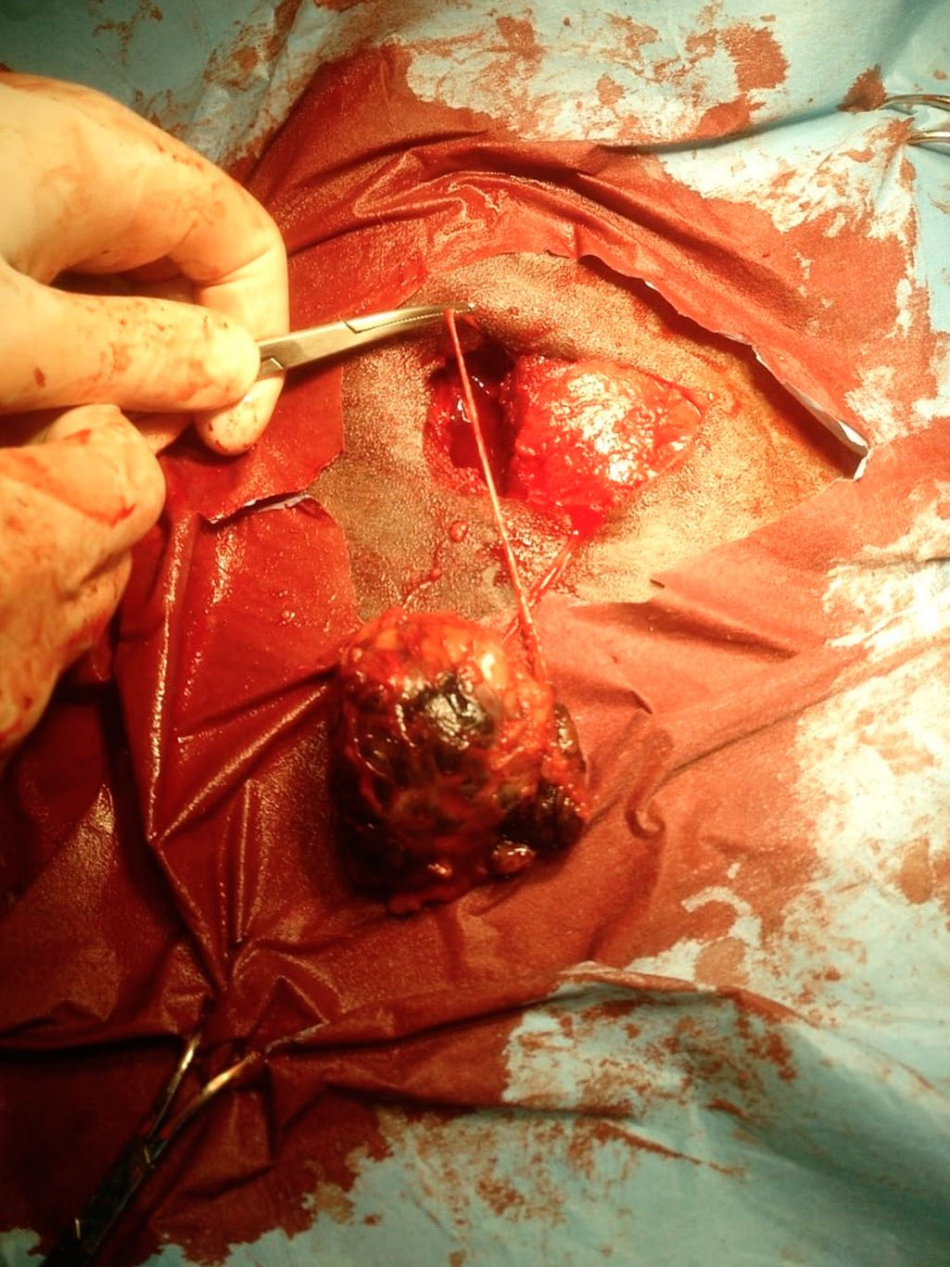
Surgical excision of the thoracic mass, from the cutaneous trunci muscle

Regarding the thoracic mass, a 5‐cm vertical incision was made over the tumour and through the skin and subcutaneous tissue, using a No 10 surgical blade. Blunt dissection of the subcutaneous tissue and cutaneous trunci muscle was performed to expose tumour margins. The mass and additional 2 cm of the surrounding musculature were sharply excised (Figure 2). The underlying thoracic serratus ventralis muscle was only minimally traumatised from the tumour resection. A penrose drain was placed into the muscle bead, exiting in the most dependent location. The surgical site was closed in a three‐layer fashion. The cutaneous trunci muscle and the subcutaneous tissue were separately sutured in a simple continuous pattern using absorbable polyglactin suture No0 on a reverse cutting needle. The skin was closed in a simple interrupted pattern with non‐absorbable polypropylene No0 suture material. The thoracic mass and five nodules from the perianal region were fixed in 10% formalin saline and submitted for histopathological examination.

Postoperatively, the gelding received phenylbutazone 3 mg/kg p.o. q. 24 hr and penicillin 20.000 IU/kg i.m. q. 24 hr in combination with streptomycin 15mg/kg i.m. q. 24 hr, for 5 days. Additionally, wound lavage with diluted Chlorhexidine solution followed by local application of silver sulfadiazine cream, was repeated twice daily for 10 days. The penrose drain was removed five days and the non‐absorbable sutures 15 days postoperatively.

## 5 RESULTS

The horse recovered uneventfully and was able to defecate normally. Gradually, the gelding returned to its normal training regime, 1 month after surgery.

The same group of veterinary surgeons that performed the surgery conducted all the subsequent examinations and diagnostic procedures. Up until 3 weeks post‐surgery, the excision sites appeared smooth, without exudate leakage or swelling.

During the first 6 months, the horse was examined on a monthly basis. The thorax was examined visually, by palpation and ultrasonography. The perianal region and the whole body of the horse were examined visually and by palpation. There were no signs of tumour regrowth at the surgical sites, neither at the thorax nor at the perianal region. No further melanocytic tumours were formed at any other site of the horse's body.

For the next 2.5 years, the horse was examined every 6 months. Rectal examination, ultrasonography of the abdominal and thoracic cavity and endoscopic examination of the respiratory tract revealed no tumour growth internally. Externally, the horse was examined visually and by palpation. Some new melanocytic nodules were formed at the perianal region, but not at the excision sites. The excision site at the lateral thorax was additionally examined per ultrasound, revealing no tumour regrowth. No other melanocytic tumours were formed at any other site of the horse's body. Haematological a biochemical values remained within normal limits.

**FIGURE 3:**
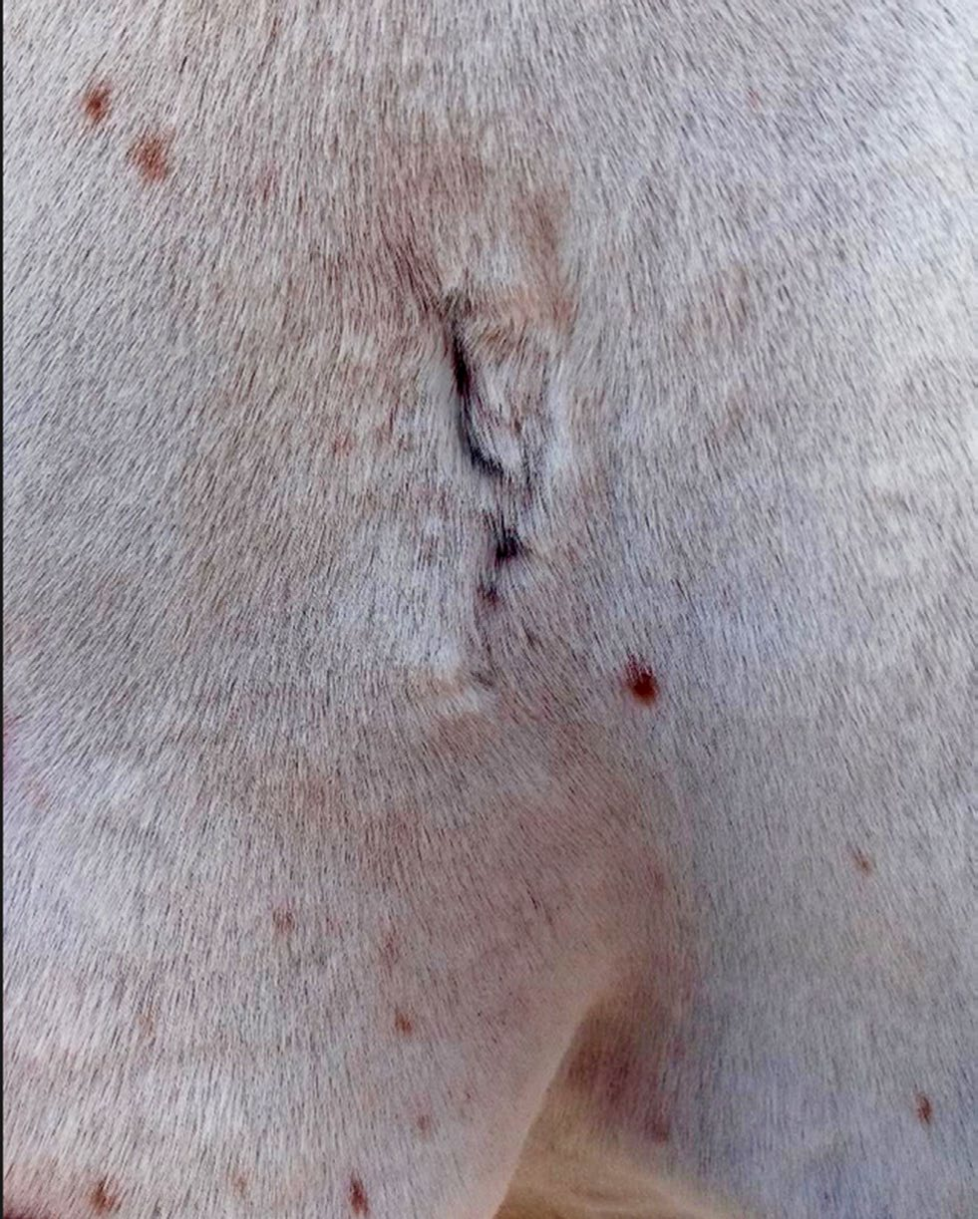
Three years post‐surgery the excision site appeared smooth with no gross evidence of tumour regrowth

So, in total, a 3‐year follow‐up revealed no gross evidence of tumour reoccurrence (Figure 3). Clinical and rectal examination as well as ultrasonography of the abdominal and thoracic cavity and endoscopic examination of the respiratory tract showed no signs of new growth formation.

## 6 HISTOPATHOLOGY

Skin and thoracic mass speciments were routinely processed and 4‐mm thick tissue sections were stained with Hematoxylin and Eosin.

**FIGURE 4:**
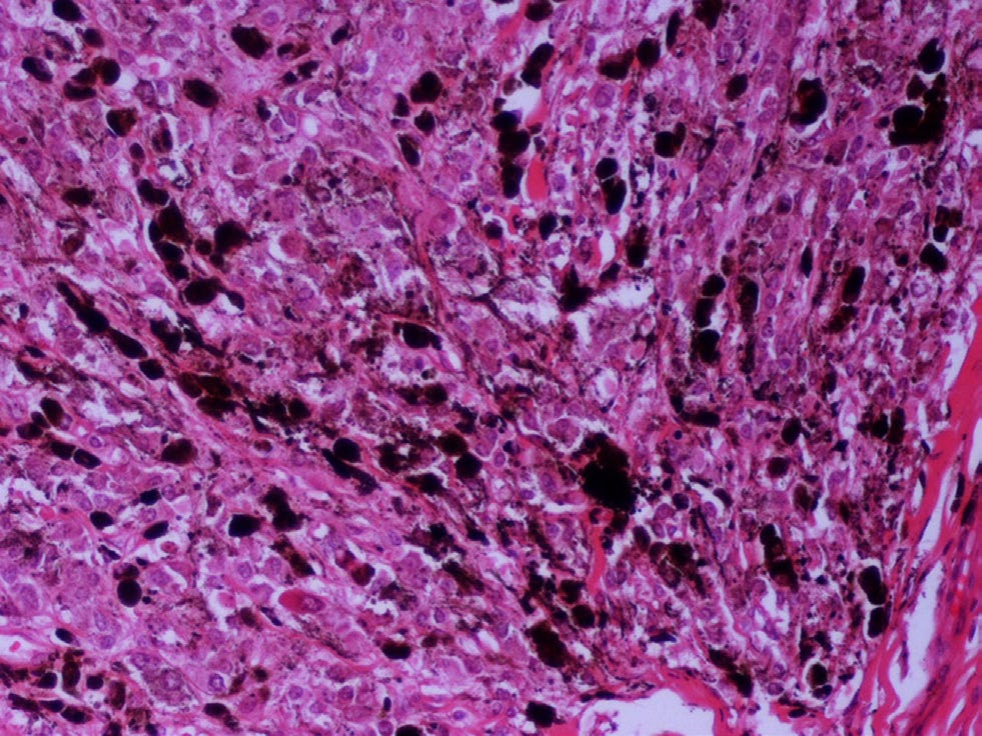
Muscle mass: numerous densely arranged melanocytes have replaced the muscle tissue. The neoplastic cells show pleomorphism and melanin granules vary from absent to abundant. Segment of degenerated muscle fibre is obvious (middle right or arrow). Original magnification ×20

Histopathologically, the thoracic mass exhibited characteristic features of malignant melanoma. Many tumour cells were pleomorphic, most of them being round or polygonal with cytoplasmic melanin granules of various degrees, although some had abundant eosinophilic cytoplasm (Figure 4). The majority of the cells showed large round euchromatic or even cystic nuclei, containing a single or two, usually large, nucleoli with eccentric localization or located adjacent to the nuclear membrane. Within certain nuclei a round eosinophilic formation was present. There were identifiable lobules composed of epitheliod or polygonal melanocytic cells, partly separated by connective tissue and skeletal muscle fibres. Mitotic figures varied from four to five per 10 high power fields (×400). A few tumour cells infiltrated the epimysial fascia that surrounded the muscle mass. According to Moore's classification system for equine malignant melanoma the thoracic mass is graded as stage four.

**FIGURE 5:**
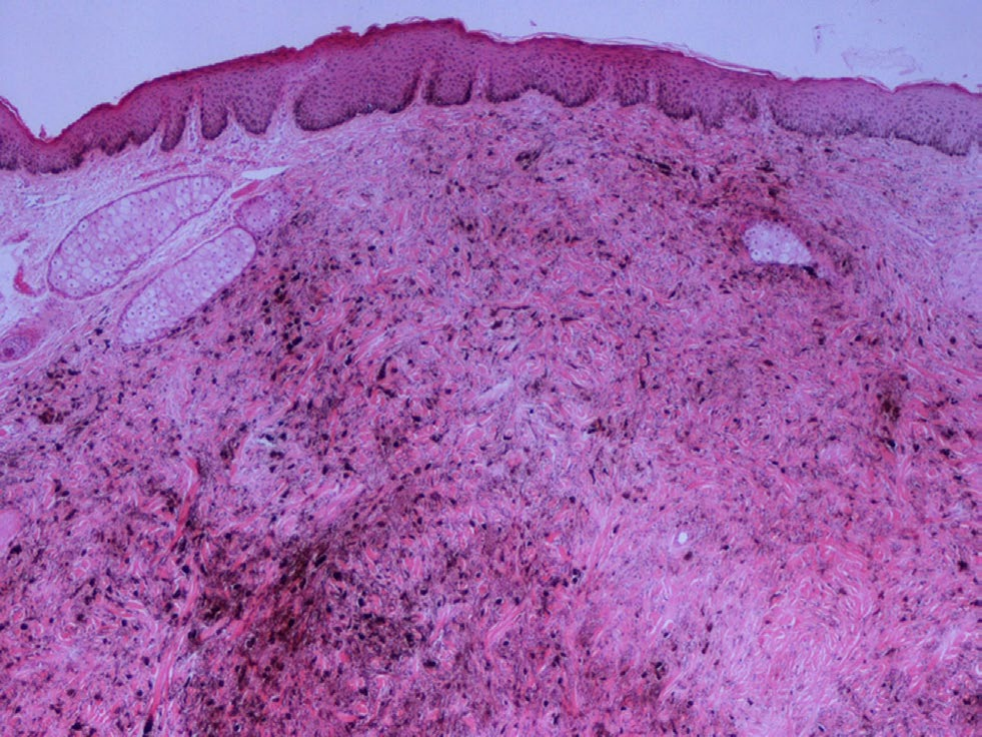
Skin nodule: melanocytes intermingled with collagen fibres, extend from the superficial dermis to its deeper layers. Tumour cells are mostly fusiform, with variable degree of cytoplasmic melanin. The epidermis shows hyperplasia. Original magnification ×4

Histopathology of the nodules excised from the perianal region, were compatible with that of dermal melanomatosis of low‐grade malignancy. The two excised specimens contained a total of five, small, intradermal, circumscribed, non‐encapsulated nodules, two of which showed a tendency to coalesce. The nodular formations consisted mainly of spindle/fusiform cells with cytoplasmic melanin granules (Figure 5). Only a small number of those cells were round in shape, with a clear cytoplasm and with few or non‐melanin granules and round to ovoid nucleus. Mitotic figures were less than three per ten high power fields ×400. Tumour cell aggregates were separated by connective tissue rich in collagen fibres. According to Moore's classification, perianal nodules were graded as stage three.

## 7 DISCUSSION

Melanocytic tumours are reported to account for 6%–15% of all equine cutaneous tumours and up to 34% of total neoplasms in horses (Knottenbelt, 2015). Although, at the time of presentation, the majority of cutaneous melanomas are recognized as histologically benign tumours, some have the tendency to eventually metastasize and thus should be considered as potentially malignant (Johnson, 1998; MacGillivray et al., 2002). According to their clinical and histopathology features, Valentine (1995) classified equine cutaneous melanocytic tumours into four distinct clinical syndromes: (a) melanocytic nevus (melanocytoma), (b) anaplastic melanoma, (c) dermal melanoma and (d) dermal melanomatosis. Melanocytic nevi are benign, solitary, superficial masses that occur in both grey and non‐grey horses and in which surgical excision is considered curative. Anaplastic malignant melanoma is rare and usually occurs in non‐grey horses older than twenty years of age. Both dermal melanoma and dermal melanomatosis are histologically similar but differ in their clinical behaviour; the first is recognized as one to two discrete masses, while the second as many coalescent lesions (Knottenbelt, 2015). It seems equally likely though, that dermal melanoma and dermal melanomatosis exist as a continuum (Moore et al., 2012).

The horse in this case report had multiple, small, single and coalescent nodules at the perianal skin and ventral surface of the tail, as well as a discrete, large, solitary mass at the lateral thorax. Based on signalment, gross appearance, location and histological examination, a definitive diagnosis of dermal melanomatosis with malignant metastasis in skeletal muscle of the thorax was made.

Tumours classified as dermal melanomatosis commonly occur at the skin of the ventral tail, the perineal region, the external genitalia and udder, ip, and periocular or parotid gland regions and are probably associated with visceral metastasis (Valentine, 1995). It is not uncommon for melanomas to give metastasis in skeletal muscles. In a retrospective study, MacGillivary et al. reported that 14% of the presented cutaneous melanomas, classified as dermal melanomas/melanomatosis, metastasized in 1–6 years. Post‐mortem examination revealed several sites of metastasis, including masses in skeletal muscles that corresponded to 57% of the examined population. In another case report, melanocytic masses causing pelvic limb lameness were discovered during necropsy in the pelvic region. Additional to other tissues affected, melanin‐filled cells had also invaded the gluteal muscle (Kirker‐Head et al., 1985).

Although melanoma metastasis to skeletal muscle is not considered to be rare, according to the authors’ knowledge, this is the first case report describing a successful surgical excision of a metastatic malignant melanoma, located in skeletal muscle of the lateral thorax. In previously published data, skeletal muscle metastases were either postmortem findings in horses with disseminated melanoma (Kirker‐Head et al., 1985; MacGillivray et al., 2002) or were melanocytic tumours located away from the primary tumour sites, though left untreated (Groom & Sullins, 2017). In the current report, the metastatic lesion was detected on a living horse and was resected successfully. Additionally, this horse showed nrither any clinical signs secondary to the specific tumour's location, nor was there any internal melanomatosis confirmed. There is currently no consensus on whether multiple lesions on a single horse are metastatic or arise spontaneously as multicentric, separate neoplasms. However, there are many cases that provide evidence of implantation, lymphatic and haematogenous metastasis (Moore et al., 2012). The authors’ of this report considered either the ventral tail or the perianal skin as a primary tumour site, where many single and coalescent melanocytic nodules were detected. It is most likely that the tumour spread to the thoracic musculature was either via blood or lymphatic circulation.

Various available treatment modalities have been suggested regarding melanocytic tumours, including cimetidine (Goetz et al., 1990), carbon dioxide laser (McCauley et al., 2002), surgical excision (Rowe & Sullins, 2004), chemotherapy (Hewes & Sullins, 2006), cisplatin (Théon et al., 2007), intratumoural injections of human interleukin‐12 (Finocchiaro et al., 2009), immunotherapy (Müller et al., 2011) and plasmid DNA vaccine encoding Streptococcus Pyogenes EMM55 protein (Brown et al., 2014). Regarding surgical excision, Rowe and Sullins (2004) reported a study of 11 cases, in which melanocytic tumours involving the perineal, perianal or perirectal region or ventral surface of the tail were surgically removed. None of these horses had evidence of tumour recurrence at the surgical site, or showed any clinical signs of internal metastasis. In a more recent study, Groom and Sullins (2017) claimed that surgical excision of large (≥4 cm) single and coalescing, perianal, melanocytic tumours was locally curative of the excised tumours. In the later report, although distant neoplasms continued to expand and some new masses were formed at remote sites, there were no cases of tumour regrowth at the excision site. In both studies, all tumours had been classified as dermal melanomatosis and dermal melanoma. Based on these data, in the present case report, surgical excision was chosen as treatment of the horse's perianal lesions. Only those tumours causing physical obstruction were removed. CO_2_ laser was used to evaporate tumour masses affecting the tail base. Laser therapy was considered to be more beneficial for the particular site, as it allows precise dissection and evaporation of tissue with minimal haemorrhage (Palmer, 2002).

Despite the fact that there is no previously published study proposing surgical removal of distant malignant metastatic melanocytic masses, surgical excision of the tumour located at the thoracic musculature was elected as the treatment of choice. Surgical margins were determined based on gross appearance of the surrounding tissue and not on histological examination. The mass was well demarcated and at least 2cm of the surrounding tissue was additionally removed. No cisplatin‐impregnated beads were implanted into the tumour bed, as complete excision of the mass was possible.

Follow‐up information was obtained via direct examination of the horse by the authors. During a period of 3 years, on a 6‐month basis, rectal examination, ultrasonography of the abdominal and thoracic cavity and endoscopic examination of the respiratory tract revealed no tumour growth internally. With respect to limitations of this case report, it was chosen not to perform exploratory laparotomy or laparoscopy in order to rule out internal metastasis, as there were no clinical grounds to do so, or was there an owner's consent as the horse appeared healthy. After 9 months, new melanocytic nodules had formed at the perianal region, but not at the excision sites. Additional ultrasonography of the thoracic musculature, showed no tumour regrowth at the removal site. No other melanocytic tumours were formed at any other site of the horse's body.

## 8 CONCLUSION

As mentioned above, according to the authors’ knowledge, this is the first case report describing a successful surgical excision of a large (6 cm), malignant, metastatic, melanocytic mass from a skeletal muscle of the lateral thorax. Equine melanomatosis commonly affects grey horses and many therapies have been suggested regarding the prevention and cure of this disease. Still, none is uniformly accepted. Treatment modalities concerning melanocytic tumours and in particular distant malignant metastatic masses need to be further studied and investigated. This report proposes surgical excision as a treatment option for distant malignant metastatic melanocytic masses in skeletal muscle.

## ETHICAL ANIMAL RESEARCH

The client‐owned animal was treated by routine surgery after informing the client of inherent risks. The client consented to surgery and histopathological diagnosis of tissue recovered from the animal.

## CONFLICT OF INTERESTS

No conflict of interests has been declared.

## AUTHORSHIPS

N. Diakakis, P. Tyrnenopoulou, T. Billi and V. Karadima performed the surgery. G.D. Brellou and E. Apostolopoulou performed histopathological specimen interpretation. All authors contributed to the writing of the manuscript.

## AUTHOR CONTRIBUTION

**Theodora Billi:** Data curation; Investigation; Visualization; Writing‐original draft; Writing‐review & editing. **Vasiliki Karadima:** Data curation; Investigation; Visualization. **Panagiota Tyrnenopoulou:** Data curation. **Emmanouela P. Apostolopoulou:** Methodology. **Georgia Brellou:** Data curation; Methodology. **NIKOLAOS DIAKAKIS:** Conceptualization; Project administration; Supervision; Validation; Visualization.

### PEER REVIEW

The peer review history for this article is available at https://publons.com/publon/10.1002/vms3.366.
